# 浸润性肺腺癌*EGFR*、*ALK*基因突变状态与影像学、病理学特征的相关性

**DOI:** 10.3779/j.issn.1009-3419.2022.101.10

**Published:** 2022-03-20

**Authors:** 赫 杨, 子成 刘, 鸿亚 王, 亮 陈, 俊 王, 伟 闻, 心峰 徐, 全 朱

**Affiliations:** 210029 南京，南京医科大学第一附属医院/江苏省人民医院胸外科 Department of Thoracic Surgery, Jiangsu Provincial People's Hospital, The First Affiliated Hospital of Nanjing Medical University, Nanjing 210029, China

**Keywords:** 肺肿瘤, 表皮生长因子受体, 间变性淋巴瘤激酶, 病理特征, 放射学, Lung neoplasms, Epidermal growth factor receptor, Anaplastic lymphoma kinase, Pathological features, Radiology

## Abstract

**背景与目的:**

当前针对肺腺癌表皮生长因子受体(epidermal growth factor receptor, *EGFR*)与间变性淋巴瘤激酶(anaplastic lymphoma kinase, *ALK*)基因突变位点的靶向治疗研究进展十分迅速，为晚期肺腺癌患者的治疗带来新的希望，然而目前*EGFR*、*ALK*基因突变在腺癌患者中所具有的特异性影像学与病理学特征仍存在较多争议，本研究进一步探索浸润性肺腺癌*EGFR*、*ALK*基因突变与影像学、病理学特征的相关性。

**方法:**

纳入2018年1月-2019年12月在我们中心接受手术的525例肺腺癌患者。根据术后基因检测的结果将患者分为*EGFR*基因、*ALK*基因突变组与野生组，并将*EGFR*基因突变组分层为外显子19、外显子21突变亚型，将突变组与野生组的病理学特征如组织学亚型、淋巴结转移、脏层胸膜侵犯(visceral pleural invision, VPI)等，影像学特征如肿瘤最大直径、实性成分占比(consolidation tumor ratio, CTR)、分叶征、毛刺征、胸膜牵拉征、空气支气管征、空泡征等进行单因素分析及多因素逻辑回归分析，探讨基因突变组是否具有特异性表现。

**结果:**

*EGFR*基因突变组常见于女性(OR=2.041, *P*=0.001)，存在更多胸膜牵拉征征象(OR=1.506, *P*=0.042)，病理学上与淋巴结转移及VPI相关性较小(*P* > 0.05)。其中外显子21突变型腺癌多见于年龄相对较大者(OR=1.022, *P*=0.036)及女性(OR=2.010, *P*=0.007)，常伴有较大肿瘤直径(OR=1.360, *P*=0.039)及胸膜牵拉征(OR=1.754, *P*=0.029)。外显子19突变型腺癌常见于女性(OR=2.230, *P*=0.009)、肿瘤实性成分比例较高(OR=1.589, *P*=0.047)、存在更多分叶征(OR=2.762, *P*=0.026)。*ALK*基因突变易发生于有吸烟史(OR=2.950, *P*=0.045)及较年轻患者(OR=1.070, *P*=0.002)，病理学上存在更多微乳头型成分(OR=4.184, *P*=0.019)及VPI(OR=2.986, *P*=0.034)。

**结论:**

*EGFR*、*ALK*基因突变型腺癌具有特异的影像学及临床病理学特征，且*EGFR*外显子19或21突变具有不同的影像学特征，对制定肺结节临床处理策略具有重要意义。

## Abstract (en)

**Background and objective:**

At present, the research progress of targeted therapy for epidermal growth factor receptor (*EGFR*) and anaplastic lymphoma kinase (*ALK*) gene mutations in lung adenocarcinoma is very rapid, which brings new hope for the treatment of advanced lung adenocarcinoma patients. However, the specific imaging and pathological features of *EGFR* and *ALK* gene mutations in adenocarcinoma are still controversial. This study will further explore the correlation between *EGFR*, *ALK* gene mutations and imaging and pathological features in invasive lung adenocarcinoma.

**Methods:**

A total of 525 patients with lung adenocarcinoma who underwent surgery in our center from January 2018 to December 2019 were included. According to the results of postoperative gene detection, the patients were divided into *EGFR* gene mutation group, *ALK* gene mutation group and wild group, and the *EGFR* gene mutation group was divided into exon 19 and exon 21 subtypes. The pathological features of the mutation group and wild group, such as histological subtype, lymph node metastasis, visceral pleural invasion (VPI) and imaging features such as tumor diameter, consolidation tumor ratio (CTR), lobulation sign, spiculation sign, pleural retraction sign, air bronchus sign and vacuole sign were analyzed by univariate analysis and multivariate *Logistic* regression analysis to explore whether the gene mutation group had specific manifestations.

**Results:**

*EGFR* gene mutation group was common in women (OR=2.041, *P*=0.001), with more pleural traction sign (OR=1.506, *P*=0.042), and had little correlation with lymph node metastasis and VPI (*P* > 0.05). Among them, exon 21 subtype was more common in older (OR=1.022, *P*=0.036), women (OR=2.010, *P*=0.007), and was associated with larger tumor diameter (OR=1.360, *P*=0.039) and pleural traction sign (OR=1.754, *P*=0.029). Exon 19 subtype was common in women (OR=2.230, *P*=0.009), with a high proportion of solid components (OR=1.589, *P*=0.047) and more lobulation sign (OR=2.762, *P*=0.026). *ALK* gene mutations were likely to occur in younger patients (OR=2.950, *P*=0.045), with somking history (OR=1.070, *P*=0.002), and there were more micropapillary components (OR=4.184, *P*=0.019) and VPI (OR=2.986, *P*=0.034) in pathology.

**Conclusion:**

The *EGFR* and *ALK* genes mutated adenocarcinomas have specific imaging and clinicopathological features, and the mutations in exon 19 or exon 21 subtype have different imaging features, which is of great significance in guiding the clinical diagnosis and treatment of pulmonary nodules.

由于早期肺癌筛查计划在全国的开展以及2019冠状病毒病(coronavirus disease 2019, COVID-19)的大流行，胸部低剂量螺旋计算机断层扫描(computed tomography, CT)筛查使得大量以磨玻璃结节为主要特征的早期肺癌患者被发现，同时肺癌作为目前全球发病率第二高的癌症(仅次于乳腺癌)，其死亡率仍居所有癌症首位^[1]^，肺癌的机制与治疗一直是近年来研究热点。

早期肺癌在影像学上往往表现为磨玻璃结节影(ground glass opacity, GGO)，而实性成分的存在被认为对肺腺癌的恶性程度及预后有较高的预测价值^[2]^。目前认为，肺腺癌的预后与组织学类型密切相关，贴壁型为主的腺癌为低复发风险，腺泡型和乳头型为主的腺癌为中等复发可能的病理亚型，而微乳头型与实体型腺癌则为有高复发风险的病理亚型^[3]^，由此可知腺癌的影像学与病理学特征决定着患者的个体化诊治方式。

当前，手术切除仍是治疗肺腺癌的主要选择，然而，随机临床试验已经证明在晚期非小细胞肺癌(non-small cell lung cancer, NSCLC)中，使用表皮生长因子受体(epidermal growth factor receptor, *EGFR*)酪氨酸激酶抑制剂(tyrosine kinase inhibitors, TKIs)治疗后的无进展生存期比化疗后更长^[4]^。EGFR-TKIs是最先应用于临床的靶向药物，是NSCLC靶向治疗的一座里程碑。此后，间变性淋巴瘤激酶(anaplastic lymphoma kinase, *ALK*)、*KRAS*等肺癌相关基因也逐渐被发现，并且有大量研究在探讨他们在NSCLC患者中的治疗潜力。*EGFR*与*ALK*基因突变为肺癌发生发展的重要危险因素^[5]^，是当前NSCLC靶向治疗的主要位点，已有较多研究探讨*EGFR*、*ALK*基因突变所具有的影像学或病理学特征，但仍存在较多争论。同时，由于国内外尚缺乏单发腺癌所含各种病理亚型成分与基因突变及突变亚型的相关性研究，且关于*EGFR*、*ALK*突变型肺腺癌的影像学表现的研究多为小样本回顾性研究，暂无统一的度量标准和分析软件，使得肺腺癌的影像学、病理学特征与基因突变的相关性未得到足够评估。因此，本研究旨在通过大样本的回顾性研究，探讨*EGFR*及*ALK*基因突变患者的临床病理学特征以及影像学特征，以期提高肺腺癌患者的诊断效能及治疗效能。

## 1 资料与方法

### 1.1 临床资料

收集2018年1月-2019年12月于南京医科大学第一附属医院行胸腔镜下肺叶切除术和亚肺叶切除术的NSCLC患者。纳入标准：①患者术前于我院行薄层胸部CT检查；②患者行单发病灶肺叶、肺段切除术或肺楔形切除术；③术后病理为浸润性腺癌(病理学分型采用2015年世界卫生组织肺部肿瘤分类)；④术后行常规肺癌相关基因检测(*EGFR*、*ALK*基因等)。排除标准：①术前行新辅助治疗的患者；②病理资料(包含淋巴结、胸膜侵犯情况及腺癌中各种组织学亚型成分所占百分比)不完善的患者；③影像学资料不清晰的患者。研究内容包括患者基本信息、影像学数据及病理学资料。本研究术后病理分期采用第8版NSCLC肿瘤原发灶-淋巴结-转移(tumor-node-metastasis, TNM)分期。

### 1.2 影像学评估

所有患者于我院行薄层胸部CT平扫检查，由2位有经验的影像医师和1位胸外科医师分别在我院影像归档和通信系统(picture archiving and communication systems, PACS)上阅片，记录肿瘤最大直径、实性成分最大直径、实性成分占比(consolidation tumor ratio, CTR)、分叶征、毛刺征、胸膜牵拉征、支气管充气征、空泡征，如遇结论不一致时，由上级医师进行分析进一步明确结果。

### 1.3 病理结果和肺癌相关基因检测

所有肿瘤组织标本于术后行福尔马林固定，石蜡包埋切片后行肺癌相关基因检测。基因检测均由我院病理科使用扩增受阻突变系统(amplification refractory mutation system, ARMS)进行荧光定量聚合酶链式反应(polymerase chain reaction, PCR)。通过查阅患者病理资料，收集所有符合纳入标准的患者的石蜡切片病理结果以及*EGFR*、*ALK*等基因突变信息。当肺腺癌中每种组织学成分(腺泡型、贴壁型、乳头型、微乳头型、实体型)比重大于5%时，该成分的存在将被记录，并以5%的增量半定量地记录各种组织类型的百分比，选择占比最大的亚型作为主要组织学亚型。

### 1.4 统计学方法

数据采用SPSS 23.0进行统计分析。符合正态分布的计量资料以均数±标准差表示，使用独立样本*t*检验分析组间差异，计数资料组间差异使用卡方检验或*Fisher*精确检验。通过建立多因素逻辑回归模型，纳入单变量分析中*P* < 0.1的变量进行多变量分析，并计算优势比(odds ratio, OR)，以*P* < 0.05为差异有统计学意义。

## 2 结果

### 2.1 基因突变组与野生组基线资料比较

在本研究纳入的525例患者中，女性405例，男性120例，平均年龄(57.93±11.07)岁，肿瘤的平均直径为(1.89±0.94)cm，包含56例(10.7%)纯磨玻璃结节，326例(62.1%)混合磨玻璃结节，143例(27.2%)为纯实性结节，478例(91.0%)患者病理TNM分期为Ⅰ期。

*EGFR*基因突变患者在所有患者中占比为59.8%(314/525)，女性患者*EGFR*突变率明显高于男性(83.6% *vs* 49.2%, *P* < 0.01)，*ALK*基因突变组患者在所有患者中占比为3.2%(17/525)，突变组患者年龄较小(*P*=0.002)，吸烟率高于野生组(*P*=0.039)(表 1)。

**表 1:** *EGFR*、*ALK*基因组基线数据及影像学、病理学特征对比 Comparison of baseline data and imaging and pathological features of patients with *EGFR* and *ALK* gene mutation group and wild group

Characteristics	*EGFR* mutation status		OR	*P*	*ALK* mutation status		OR	*P*
	Yes (*n*=314)	No (*n*=211)			Yes (*n*=17)	No (*n*=508)		
Age (yr)	58.65±10.81	56.86±11.38		0.069	49.77±13.58	58.20±10.89		0.002
Gender [*n* (%)]			0.569	0.007			1.424	0.718
Male	59 (18.8)	61 (28.9)			5 (29.4)	115 (22.6)		
Female	255 (81.2)	150 (71.1)			12 (70.6)	393 (77.4)		
Smoking history [*n* (%)]			0.625	0.055			3.303	0.039
Yes	39 (12.4)	39 (18.5)			6 (35.3)	72 (14.2)		
No	275 (87.6)	172 (81.5)			11 (64.7)	436 (85.8)		
BMI (mg/m^2^)	23.45±3.32	23.36±2.74		0.737	22.70±4.01	23.44±3.07		0.337
TNM [*n* (%)]				0.085				0.130
Ia+Ib	282 (89.8)	196 (92.9)			14 (82.4)	464 (91.3)		
IIa+IIb	16 (5.1)	12 (5.7)			1 (5.9)	27 (5.3)		
IIIa	16 (5.1)	3 (1.4)			2 (11.7)	17 (3.4)		
Radiological features								
Tumor diameter (cm)	1.98±0.93	1.75±0.95		0.008	1.98±1.68	1.89±0.91		0.817
Solid component diameter (cm)	1.28±1.01	1.03±0.95		0.004	1.60±1.87	1.16±0.95		0.356
CTR	0.61±0.34	0.55±0.35		0.052	0.72±0.36	0.58±0.34		0.114
CTR≥0.5 [*n* (%)]	196 (62.4)	110 (52.1)	1.525	0.019	12 (70.6)	294 (57.9)	1.747	0.296
Lobulated border [*n* (%)]	289 (92.0)	182 (86.3)	1.842	0.032	14 (82.4)	457 (90.0)	0.521	0.542
Spiculated margin [*n* (%)]	304 (96.8)	205(97.2)	0.890	0.824	16 (94.1)	493 (97.0)	0.487	0.414
Air bronchogram [*n* (%)]	159 (41.1)	102 (48.3)	1.096	0.606	6 (35.3)	255 (50.2)	0.541	0.227
Pleural retraction [*n* (%)]	207 (65.9)	109 (51.7)	1.810	0.001	10 (58.8)	306 (60.2)	0.943	0.907
Bubble-like lucency [*n* (%)]	53 (16.9)	43 (20.4)	0.793	0.309	5 (29.4)	91 (17.9)	1.909	0.375
Histology subtype [*n* (%)]								
Lepidic	192 (61.1)	138 (65.4)	0.833	0.322	5 (29.4)	325 (64.0)	0.235	0.004
Acinar	283 (90.1)	177 (83.9)	1.754	0.033	14 (82.4)	446 (87.8)	0.649	0.767
Papillary	92 (29.3)	47 (22.3)	1.446	0.074	4 (23.5)	135 (26.6)	0.850	> 0.999
Micropapillary	23 (7.3)	12 (5.7)	1.311	0.461	5 (29.4)	30 (5.9)	6.639	0.001
Solid	33 (10.5)	22 (10.4)	1.009	0.976	2 (11.8)	53 (10.4)	1.145	> 0.999
LN metastasis [*n* (%)]	20 (6.4)	10 (4.7)	1.367	0.430	2 (11.8)	28 (5.5)	2.286	0.253
VPI [*n* (%)]	30 (9.6)	13 (6.2)	1.609	0.165	5 (29.4)	38 (7.5)	5.154	0.005
CTR: consolidation tumor ratio; LN: lymph nodes; VPI: visceral pleural invasion; EGFR: epidermal growth factor receptor; ALK: anaplastic lymphoma kinase; BMI: body mass index; TNM: tumor-node-metastasis.								

### 2.2 *EGFR*、*ALK*基因突变组与野生组的影像学特征比较

根据薄层胸部CT影像，*EGFR*基因突变组在胸部CT轴位图像上的肿瘤直径、实性成分直径大于野生组(分别为*P*=0.008和*P*=0.004)，在CTR上无显著差异(*P*=0.052)，但突变组在CTR≥0.5上占比大于野生组(*P*=0.019)(表 1)。进一步分层分析显示：外显子21突变型腺癌直径大于野生型(*P*=0.034)，外显子19突变型腺癌的实性成分直径大于野生型(*P*=0.019)，外显子19突变型腺癌在CTR及CTR≥0.5占比上大于野生型及外显子21突变型(*P*=0.001)(表 2)。形态学上，突变组较野生组存在更多分叶征(*P*=0.032)、胸膜牵拉征(*P*=0.001)。

**表 2:** *EGFR*基因突变亚型分层分析及组内对照分析 Stratification analysis and intra-group control analysis of *EGFR* gene mutation subtype

Characteristics	*EGFR* wild type (*n*=211)	*EGFR* mutated		*P*
		Exon 19 (*n*=111)	Exon 21 (*n*=176)	
Age (yr)	56.86±0.78	57.13±1.05	60.19±0.77	0.007^b, c^
Gender [*n* (%)]				0.023^a, c^
Male	61 (28.9)	21 (18.9)	32 (18.2)	
Female	150 (71.1)	90 (81.1)	144 (81.8)	
Smoking history [*n* (%)]				0.146
Yes	172 (81.5)	95 (85.6)	156 (88.6)	
No	39 (18.5)	16 (14.4)	20 (11.4)	
Radiological characteristics				
Tumor diameter (cm)	1.76±0.07	1.86±0.08	2.00±0.07	0.034^c^
Solidcomponent diameter (cm)	1.03±0.07	1.34±0.09	1.20±0.08	0.019^a^
CTR	0.55±0.02	0.69±0.03	0.55±0.03	0.001^a, b^
CTR≥0.5 [*n* (%)]	110 (52.1)	83 (74.8)	96 (54.5)	0.001^a, b^
Lobulated border [*n* (%)]	182 (86.3)	104 (93.7)	163 (92.6)	0.041^a, c^
Spiculated margin [*n* (%)]	205 (97.2)	106 (95.5)	172 (97.7)	0.550
Pleural retraction [*n* (%)]	109 (51.7)	73 (65.8)	116 (65.9)	0.006^a, c^
Air bronchogram [*n* (%)]	102 (48.3)	54 (48.6)	93 (52.8)	0.644
Bubble-like lucency [*n* (%)]	43 (20.4)	22 (19.8)	27 (15.3)	0.409
Histological subtype [*n* (%)]				
Lepidic	138 (65.4)	62 (55.9)	117 (66.5)	0.149
Acinar	177 (83.9)	102 (91.9)	158 (89.8)	0.068
Papillary	47 (22.3)	36 (32.4)	42 (23.9)	0.122
Micropapillary	12 (5.7)	10 (9.0)	10 (5.7)	0.453
Solid	22 (10.4)	10 (9.0)	20 (11.4)	0.817
LN metastasis [*n* (%)]	10 (4.7)	9 (8.1)	8 (4.5)	0.365
VPI [*n* (%)]	13 (6.2)	11 (9.9)	15 (8.5)	0.450
^a^: *EGFR* wild type group *vs* exon 19 mutation group; ^b^: *EGFR* exon 19 mutation group *vs* exon 21 mutation group; ^c^: *EGFR* wild type group *vs* exon 21 mutation group.				

*ALK*基因突变组与野生组在上述影像学特征上无明显统计学差异(表 1)。

### 2.3 *EGFR*基因组与*ALK*基因组的病理结果比较

*EGFR*基因组在有无淋巴结转移、转移的淋巴结站点及有无脏层胸膜侵犯(visceral pleural invasion, VPI)等病理结果方面无显著差异。在组织学亚型上，突变组腺癌较野生组存在更多腺泡型成分(*P*=0.033)。

*ALK*基因组在有无淋巴结转移、转移的淋巴结站点无显著差异，突变组VPI的发生率高于野生组(*P*=0.005)，在组织学亚型上，野生型腺癌存在更多贴壁型成分(*P*=0.004)，突变型存在更多微乳头成分(*P*=0.001)(表 1)。

在肺腺癌主要组织学亚型方面，*EGFR*基因野生组多为贴壁为主型(*P*=0.014)，突变组多为腺泡为主型(*P*=0.037)，*ALK*基因突变组与野生组在主要病理学亚型方面无统计学差异(表 3)。

**表 3:** 主要组织学亚型与基因突变的关系[*n* (%)] The relationship between main histological subtypes and gene mutation [*n* (%)]

Main histological subtype	*EGFR* mutation status		OR	*P*	*ALK* mutation status		OR	*P*
	Yes (*n*=314)	No (*n*=211)			Yes (*n*=17)	No (*n*=508)		
Lepidic	85 (27.1)	77 (36.5)	0.646	0.014	2 (11.7)	160 (31.5)	0.290	0.109
Acinar	200 (63.7)	115 (54.5)	1.465	0.037	12 (70.6)	303 (59.6)	1.624	0.455
Papillary	23 (7.3)	9 (4.3)	1.774	0.193	1 (5.9)	31 (6.1)	0.877	> 0.999
Micropapillary	0 (0.0)	2 (0.9)	0	0.161	1 (5.9)	1 (0.2)	31.688	0.064
Solid	6 (1.9)	8 (3.8)	0.494	0.269	1 (5.9)	13 (2.6)	2.380	0.373

### 2.4 多因素逻辑回归分析

将上述单因素统计分析所得*P* < 0.1的变量纳入多因素逻辑回归分析，结果显示女性(OR=2.041, 95%CI: 1.330-3.134, *P*=0.001)、胸膜牵拉征(OR=1.506, 95%CI: 1.014-2.237, *P*=0.042)为*EGFR*基因突变的独立预测因素。分层分析中：年龄相对较大(OR=2.010, 95%CI: 1.001-1.044, *P*=0.036)、女性(OR=2.010, 95%CI: 1.210-3.339, *P*=0.007)、较大肿瘤直径(OR=1.360, 95%CI: 1.026-2.489, *P*=0.034)及胸膜牵拉征(OR=1.754, 95%CI: 1.048-2.915, *P*=0.029)为外显子21突变型腺癌的独立预测因素。女性(OR=2.230, 95%CI: 1.226-4.057, *P*=0.009)、CTR(OR=1.589, 95%CI: 1.012-3.456, *P*=0.047)、CTR≥0.5(OR=1.230, 95%CI: 1.125-1.369, *P*=0.038)及分叶征(OR=2.762, *P*=0.026)为外显子19突变型腺癌的独立预测因素。

在*ALK*基因研究组中，年龄相对较小(OR=1.070, 95%CI: 1.025-1.117, *P*=0.002)、有吸烟史(OR=3.937, 95%CI: 1.311-11.765, *P*=0.015)、微乳头型成分的存在(OR=4.184, 95%CI: 1.261-13.889, *P*=0.019)及VPI(OR=1.406, 95%CI: 1.034-2.345, *P*=0.034)为*ALK*基因突变的独立预测因素(表 4)。

**表 4:** 多因素逻辑回归分析 Multivariate *Logistic* regression analysis

Characteristics	*EGFR* mutated			*EGFR*-exon 19			*EGFR*-exon 21			*ALK* mutated	
	OR (95%CI)	*P*		OR (95%CI)	*P*		OR (95%CI)	*P*		OR (95%CI)	*P*
Age							1.022 (1.001-1.044)	0.036		1.070 (1.025-1.117)	0.002
Gender											
Male	Reference			Reference			Reference				
Female	2.041 (1.330-3.134)	0.001		2.230 (1.226-4.057)	0.009		2.010 (1.210-3.339)	0.007			
Smoking history											
No										Reference	
Yes										2.950 (1.022-8.475)	0.045
Tumor diameter (cm)							1.360 (1.026-2.489)	0.039			
CTR				1.589 (1.012-3.456)	0.047						
CTR≥0.5				1.230 (1.125-1.369)	0.038						
Lobulated border				2.762 (1.126-6.767)	0.026						
Pleural retraction	1.506 (1.014-2.237)	0.042					1.754 (1.048-2.915)	0.029			
Pathology type											
Micropapillary										4.184 (1.261-13.889)	0.019
VPI										1.406 (1.034-2.345)	0.034

## 3 讨论

*EGFR*、*ALK*基因是NSCLC的重要驱动基因，且对靶向治疗的选择具有决定性作用，肿瘤的影像学及病理学特征同样在腺癌的规范诊治决策中至关重要^[6]^，然而三者之间的联系尚未完全阐明，在这项回顾性研究中，我们探讨了手术切除的肺腺癌患者基因突变与影像学、病理学特征的相关性。

本研究中*EGFR*及*ALK*基因突变患者的占比分别为59.8%和3.24%，与既往研究^[7, 8]^所报道的60%和2%-7%相一致。女性患者的*EGFR*突变率较男性高(*P*=0.007)，无吸烟史患者占比较高但无显著差异(*P*=0.055)，与先前研究^[9]^所报道*EGFR*基因突变易发生于不吸烟的年轻女性基本相符。*ALK*突变患者易发生于年轻患者(*P*=0.002)，吸烟率高于野生组(*P*=0.039)，有研究^[10]^报道*ALK*重排易发生于年轻的非吸烟或少吸烟腺癌患者，其中的差异可能与本研究纳入*ALK*突变的病例数相对较少且未将吸烟程度明确分级有关，目前吸烟史与*ALK*突变之间的关系暂不明确，尚不能排除吸烟暴露是腺癌的异质性来源^[11]^。

目前研究^[12]^多表明*EGFR*突变在CT中更多地表现为GGO，亦有研究^[13]^发现*EGFR*突变与实性成分相关。本研究中*EGFR*基因突变组较野生组肿瘤拥有更大直径、实性成分，而多因素逻辑回归分析显示肿瘤大小及实性成分并非*EGFR*突变的独立预测因素。这一发现可能的解释为EGFR表达与CT上GGO的百分比呈负相关^[14, 15]^，且*EGFR*突变可以促进GGO向实性成分转化^[16]^。为更进一步探讨其相关性，本研究对在*EGFR*突变中占比约85%且对EGFR-TKI敏感的经典外显子19、外显子21突变位点^[17]^进行分层分析，结果表明外显子21突变型腺癌的肿瘤直径更大，外显子19突变型腺癌的实性成分占比更多。我们有理由认为外显子19与外显子21突变型腺癌影像学表现不同，对突变亚型分层可更精准地探讨*EGFR*突变与实性成分的相关性。

此外，本研究揭示了*EGFR*突变型腺癌在CT上更多表现为分叶征、胸膜牵拉征，且多因素逻辑回归分析显示胸膜牵拉征为*EGFR*突变的重要预测因素，这与Cheng等^[18]^的荟萃分析研究一致。分层分析进一步发现胸膜牵拉征为外显子21突变的重要预测因素，分叶征为外显子19突变的重要预测因素。胸膜牵拉征指胸膜受牵拉向肿瘤方向收缩，由肿瘤的反应性纤维化和瘢痕增生形成，分叶征则代表肿块向各个方向生长速度不一，我们的研究证实其均为腺癌诊断的重要影像学表现，研究未发现其他CT特征与*EGFR*突变之间的相关性，而Qiu等^[19]^研究发现*EGFR*突变型腺癌以分叶征、毛刺征、支气管充气征、胸膜牵拉征多见。对上述差异的解释可能是由于多种影像学征象的判断主观性强，各研究之间无统一的研究设计、评判标准。未来，影像组学利用数字图像处理技术、机器学习和统计学方法，以其定性、定量的优势精准分析肺癌的病灶形态，将进一步探究基因突变与影像学特征的相关性^[20]^。

在病理学方面，我们发现*EGFR*突变与淋巴结转移及胸膜侵犯的相关性较小，Zou等^[21]^通过多因素回归分析发现淋巴结转移在*EGFR*突变组与野生组间无明显差异，与本研究一致，Shi等^[22]^研究发现与*EGFR*野生型相比，19del和L858R突变均能显著增加NSCLC患者VPI的发生风险，Shi等^[22]^的研究对象为包含鳞癌的Ⅲ期占比为21.9%的NSCLC患者，存在VPI的患者占比较高(45.1%)，而本研究中Ⅰ期患者占比较高(91.0%)，研究的差异可能是选择偏倚导致的，但本研究表明早期腺癌*EFGR*突变与胸膜侵犯无显著相关。既往研究^[23]^表明，腺泡优势型肺腺癌*EGFR*基因突变率高于其他亚型腺癌，本研究亦证实*EGFR*突变型腺癌与腺泡型成分关系密切，且存在更多腺泡为主型腺癌。

先前研究^[24, 25]^表明*ALK*基因突变阳性患者肿瘤具有更大体积、实性肿块，本研究亦发现突变组具有较大的肿瘤直径及实性成分，可能因纳入的*ALK*突变病例较少而差异不显著(*P* > 0.05)。本研究中*ALK*基因突变组在影像学特征方面无特异性表现，Chang等^[26]^研究发现*ALK*突变组与野生组在腺癌的分叶征、毛刺、气管征、空泡征、胸膜牵拉征上无显著差异，与本研究一致。

**图 1:**
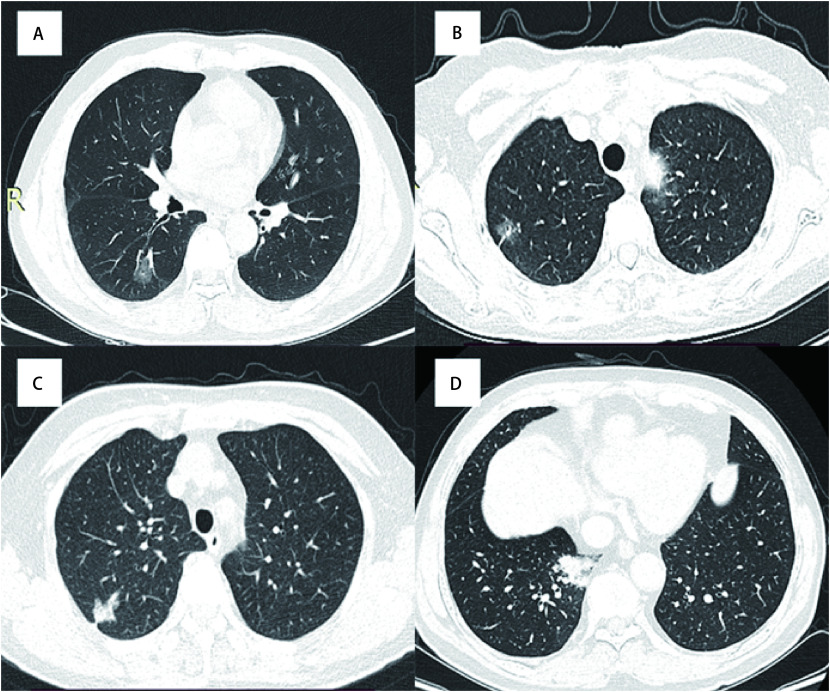
典型的CT征象。A：*EGFR*、*ALK*基因野生型腺癌，以磨玻璃成分为主；B：*EGFR*基因外显子21突变型腺癌，存在胸膜牵拉征及较多磨玻璃成分；C：*EGFR*基因外显子19突变型腺癌，存在分叶征及较多实性成分；D：*ALK*基因突变型腺癌，以实性肿块为主。 Typical CT signs. A: *EGFR* and *ALK* gene wild-type adenocarcinoma, mainly composed of ground glass; B: Exon 21 mutant adenocarcinoma had pleural traction sign and more ground glass components; C: Exon 19 mutant adenocarcinoma had lobulation sign and more solid components; D: *ALK* gene mutant adenocarcinoma is mainly solid mass. CT: computed tomography.

有研究^[27-29]^表明*ALK*突变易出现淋巴结转移及胸膜侵犯，是预后不良的一个预测因素，本研究同样证实*ALK*突变腺癌易出现胸膜侵犯，但与淋巴结转移无显著相关，这可能与本中心所纳入的*ALK*突变多为Ⅰa期(76.5%)肿瘤有关，同时我们还发现Ⅰa期周围型肺癌淋巴结转移率很低，尤其CT上以GGO为主的腺癌几乎没有发现淋巴结转移。本研究发现*ALK*基因突变患者组织学亚型中含有更多微乳头成分，多因素回归分析证实微乳头型腺癌成分为*ALK*基因突变的独立预测因素。Li等^[27]^研究发现*ALK*阳性患者术后的无病生存期明显短于*EGFR*突变阳性患者和野生型患者，且既往研究多提示*ALK*重排为较低的无病生存率的显著预测因素，我们可以推测微乳头成分的增多提高了*ALK*突变的概率，同时降低了患者的生存率。

本研究亦存在一些局限：这些数据是基于单中心的回顾性研究，不可避免地会造成选择偏差，研究纳入*ALK*突变阳性的病例数相对较少且肿瘤分期较早，上述局限都是可能导致本研究结果与既往研究存在差异的原因；我们的随访时间较短，研究未纳入关于腺癌患者预后的随访数据，无法直接得出*EGFR*及*ALK*基因突变状态与预后的关系。然而，我们的研究结果为进一步的研究提供了方向。

在NSCLC患者中，女性在CT上有胸膜牵拉征的患者更容易发生*EGFR*突变且不同突变亚型影像病理学表现有差异。年轻的吸烟患者及拥有微乳头成分患者其*ALK*突变发生率更高，且其更易发生VPI。这些结果有利于临床医生将临床、影像、病理特征与驱动基因突变的表现结合起来，在临床上加快筛选潜在的驱动基因突变人群，制定最佳治疗策略，使患者接受个体化的针对性治疗而提高治疗效果，在临床诊治肺腺癌上具有重要意义。我们期待多中心、前瞻性的研究对本研究结果进行验证与拓展，为肺腺癌患者的预测评估建立精准的影像-病理-基因表型综合模型。
